# Long-term neurodevelopmental consequences of intrauterine exposure to lithium and antipsychotics: a systematic review and meta-analysis

**DOI:** 10.1007/s00787-018-1177-1

**Published:** 2018-06-11

**Authors:** Eline M. P. Poels, Lisanne Schrijver, Astrid M. Kamperman, Manon H. J. Hillegers, Witte J. G. Hoogendijk, Steven A. Kushner, Sabine J. Roza

**Affiliations:** 1 Department of Psychiatry, Erasmus University Medical Center, ’s-Gravendijkwal 230, 3015 CE Rotterdam, The Netherlands; 2 Department of Child and Adolescent Psychiatry, Erasmus University Medical Center, Rotterdam, The Netherlands

**Keywords:** Lithium, Antipsychotics, Pregnancy, Neurodevelopment, Intrauterine exposure

## Abstract

Lithium and antipsychotics are often prescribed to treat bipolar disorder or psychotic disorders in women of childbearing age. Little is known about the consequences of these medications during pregnancy for the developing child. The objective of this article is to systematically review findings from preclinical and clinical studies that have examined the neurodevelopmental consequences of intrauterine exposure to lithium and antipsychotics. A systematic search was performed in Embase, Medline, Web of Science, PsychINFO, Cochrane, and Google Scholar. Clinical and experimental studies were selected if they investigated neurodevelopment of offspring exposed to lithium or antipsychotics during gestation. Quality of clinical and preclinical studies was assessed by the Newcastle–Ottawa Scale and the SYRCLE’s risk of Bias tool, respectively. In total, 73 studies were selected for qualitative synthesis and three studies were selected for quantitative synthesis. Of preclinical studies, 93% found one or more adverse effects of prenatal exposure to antipsychotics or lithium on neurodevelopment or behaviour. Only three clinical cohort studies have investigated the consequences of lithium exposure, all of which reported normal development. In 66% of clinical studies regarding antipsychotic exposure, a transient delay in neurodevelopment was observed. The relative risk for neuromotor deficits after in utero exposure to antipsychotics was estimated to be 1.63 (95% CI 1.22–2.19; *I*^2^ = 0%). Preclinical studies suggest long-term adverse neurodevelopmental consequences of intrauterine exposure to either lithium or antipsychotics. However, there is a lack of high-quality clinical studies. Interpretation is difficult, since most studies have compared exposed children with their peers from the unaffected population, which did not allow correction for potential influences regarding genetic predisposition or parental psychiatric illness.

**Electronic supplementary material:**

The online version of this article (10.1007/s00787-018-1177-1) contains supplementary material, which is available to authorized users.

## Key points

**Table:** 

Preclinical studies suggest a harmful effect of lithium on motor activity, developmental milestones and reflexes, spatial memory and brain weight
Only three clinical cohort studies on the development of children with in utero exposure to lithium are published in the literature. They report normal development.
Most preclinical studies found a harmful effect of intrauterine exposure to antipsychotics on motor activity, developmental milestones and reflexes and spatial memory
Clinical studies suggest a delay in motor functioning of children with in utero exposure to antipsychotics. In several studies, this delay appeared to be transient

## Introduction

Patients with bipolar disorder or a psychotic disorder are often treated with lithium and/or antipsychotics in the acute phase of the disease and chronically for relapse prevention [1, 2]. As a substantial proportion of patients with bipolar disorder or a psychotic disorder are women of childbearing age, knowledge of the potentially deleterious consequences of intrauterine exposure to lithium and/or antipsychotics is critically important for optimally weighing the risks and benefits of different pharmacotherapy options. Continuation of maintenance treatment during pregnancy is often necessary to maintain symptom stability and prevent relapse, while discontinuation of lithium or antipsychotics is associated with a higher relapse risk [3–5].

The teratogenic, obstetric and neurodevelopmental consequences of intrauterine exposure to lithium and/or antipsychotics have remained poorly defined, largely due to the difficulty of implementing feasible study designs that avoid confounding by indication. Multiple studies have reported a positive association between intrauterine exposure to lithium and the risk of cardiovascular anomalies [6–10]. Lithium use during pregnancy has also been associated with an increased rate of miscarriages and preterm delivery [7, 9]. Similarly, antipsychotic use during pregnancy has been associated with higher rates of preterm delivery and low birth weight, as well as neonatal withdrawal symptoms, sedation and extrapyramidal side effects [11, 12]. However, severe mental illness, the indication for which lithium and antipsychotics are overwhelmingly prescribed during pregnancy, is also associated with increased risk of obstetric and neonatal complications independent of medication [13–15]. Therefore, confounding by indication has remained a challenging issue limiting the conclusiveness of previously observed associations between neonatal outcomes and medication exposure during pregnancy.

Further compounding the issue of study design, little is known about the long-term neurodevelopmental consequences of intrauterine exposure to lithium or antipsychotics. It is widely assumed that the fetal environment influences lifetime disease risk based on Barker’s hypothesis of ‘fetal and infant origins of adult life’ [16, 17]. Following this reasoning, adverse fetal or neonatal consequences of intrauterine exposure to lithium and/or antipsychotics might be expected to have neurodevelopmental consequences that extend well beyond infancy. Regarding the cellular mechanisms of lithium, a neuroprotective effect is suggested through inhibition of glycogen synthase kinase-3 (GSK-3) [18, 19]. Mechanisms of antipsychotic action differ between the different types of antipsychotic medication with dopamine D2 receptor antagonism as the general pharmacodynamic property [20]. Several studies have suggested that atypical antipsychotics, but not typical antipsychotics, may also have neuroprotective effects [21]. Evidence from clinical neuroimaging studies in adults suggests that the use of lithium or antipsychotic medication can influence brain structure. Structural magnetic resonance imaging (MRI) studies have shown that lithium is associated with increases or normalization of gray matter volume in fronto-limbic brain structures [22], while antipsychotic medication has been associated with decreased brain volume and increased ventricular size [23]. Based on this information one might expect similar, or even larger, effects when not the adult but the fetus is exposed during a crucial stage of neurodevelopment.

The objective of this article is to systematically review and synthesize findings drawn from both preclinical and clinical studies examining long-term neurodevelopmental outcomes following intrauterine exposure to lithium or antipsychotics, in an effort to gain further insight into the risks associated with the use of these medications during pregnancy.

## Methods

### Search strategy for systematic review

A systematic search was performed by a trained librarian in the following databases: (1) Embase, (2) MEDLINE, (3) Web of Science, (4) PsychINFO, (5) Cochrane, and (6) Google Scholar, from their respective inceptions through June 8, 2017 to identify studies that investigated the long-term neurodevelopmental consequences of intrauterine exposure to lithium or antipsychotics. The search included the following elements: lithium, antipsychotics, (neuro) development and intrauterine exposure. All elements were transformed into a thesaurus suitable for each specific database. The exact search terms per database are reported in the Supplementary material (Supplement 1).

### Study selection

Studies were considered eligible for inclusion if they were written in the English language and investigated the long-term neurodevelopment, defined as neurodevelopment beyond the newborn period, of offspring exposed to lithium or antipsychotics during gestation. Experimental preclinical investigations and clinical investigations were considered eligible for inclusion. Case reports were also included in this review. Two reviewers (EP and LS) independently screened the title and abstract of all records identified by our database search. Full text articles were obtained from the studies selected during this first screening step. Both reviewers independently selected the full text articles that met the eligibility criteria. The inclusion of both reviewers was compared and consensus was made on the final inclusion. An additional search was performed on the reference section of relevant studies and review articles to screen for other eligible articles that were otherwise not identified by our structured search.

### Data extraction

Two authors (EP and LS) independently extracted data on study design, sample size and characteristics, medication dosage and exposure period, follow-up time, and behavioural, cognitive and neurological outcome measures. The data were summarized in a data extraction form. Studies were categorized by medication type and study design, and results were reported descriptively in accordance with the PRISMA statement [24].

### Assessment of the risk of bias and the quality of studies

Methodological quality and risk of bias was assessed independently for each study by two reviewers (EP and LS). Risk of bias in preclinical studies was assessed with the SYRCLE’s risk of Bias tool [25]. This tool was recently developed to assess risk of bias and has been adjusted for specific aspects of animal intervention studies. The Newcastle–Ottawa Scale (NOS) [26] was used for clinical studies. The NOS assesses the risk of bias of observational studies based on selection, comparability and outcome criteria. The NOS rating scale varies from zero to nine; with zero representing the highest risk of bias and nine the lowest risk.

### Procedure for meta-analysis

For our meta-analysis, we used the same search strategy as mentioned before. Only clinical investigations were included in the meta-analysis with the goal to enhance further insight into the risks associated with the use of these medications during pregnancy in humans. Pooling was performed per type of neuropsychological outcome and per group of medication exposure (lithium or antipsychotics) over a minimum of two studies. Fixed and random-effect estimation was used. In case of substantial heterogeneity, a random-effect estimation provides more reliable pooled results. Results are plotted in a forest plot. Cochran’s *Q* test, and *I*^2^ statistics were used to quantify heterogeneity across trials. *I*^2^ >40% was considered as substantial heterogeneity. The influence of intrauterine exposure to lithium or antipsychotics on neuropsychological development over time was estimated using random effects meta-regression analysis. Statistical analyses were performed using the ‘Metan package’ in Stata 15 [27].

## Results

### Study selection

**Fig. 1:**
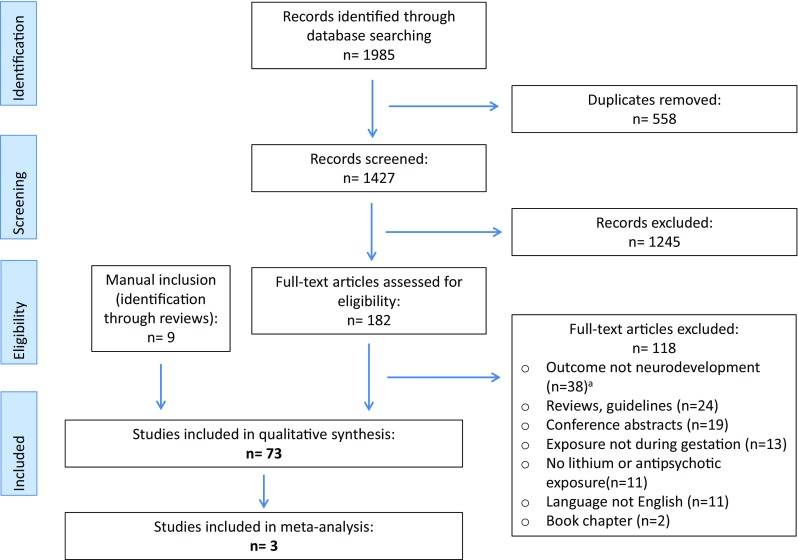
Flowchart of the study selection process in this systematic review and meta-analysis. ^a^Outcome of the excluded studies: cell development (*n* = 4), teratogenicity (*n* = 5), neonatal outcome only (*n* = 14), obstetric outcome and teratogenicity (*n* = 9), fetal development (*n* = 2), endocrine and cardiologic follow-up (*n* = 1), weight gain and mortality (*n* = 1), treatment choice (*n* = 1), sexual development (*n* = 1)

The study selection process is presented in Fig. 1. Our initial search produced a total of 1985 articles. After duplicates were removed, 1427 articles remained. Based on the screening of title and abstract, 182 full text articles were examined for eligibility, of which 118 were excluded (Fig. 1). In total, 73 studies were included in the qualitative synthesis, of which nine studies were included through manual (non-structured) identification. Additionally, three studies were included in the quantitative synthesis.

### Study characteristics

The characteristics and results of the preclinical investigations included in the qualitative analysis are summarised in Table 1 and 2. The characteristics and results of the clinical studies can be found in Table 3 and 4. Table 1 and 2 in the Supplementary Material (Supplement 2) present the characteristics and results of case reports.

**Table 1:** Characteristics and results of preclinical studies on intrauterine exposure to lithium

Author (year)	Species/strain	Lithium dosage	Control medication	Exposed period	Follow-up time	Measurements	Results
Brain (1986) [35]	Mice/SW	0.1 or 0.2 mEq s.c.	n.r.	Last 4 days of gestation until PND 4	36 days	Standard opponent test: social, defensive, threatening and aggressive behaviour	No difference
Messiha (1986) [34]	Mice/SD	Lithium 1 mEq solution	Distilled water	G1 until PND 23	37 days	Brain weight	Decrease in brain weight (8.6% female, 8.2% male)
Sechzer (1986) [29]	Rats/SD	2.0 or 4.0 mEq/kg/day in orange juice solution	Orange juice solution	G1 until PND 28	4 months	Eye and ear opening, startle response, depth perception, open field activity	Delayed eye and ear opening, startle response and maturation of depth perception. Less spontaneous activity at 4 months
Rider (1978) [33]	Rats/n.r.	15 mEq/L water	Water and low protein diet	During gestation and lactation (days not specified)	4.5–5.5 months	T-maze performance, avoidance response	Decreased performance on the T-maze, decreased avoidance response
Teixeira (1995) [32]	Rats/W	10 mM in tap water	Tap water (restricted) Tap water ad libitum	Whole gestation period or G1 until PND 21	21 days	Righting reflex, eye opening, cliff avoidance test, motor coordination (rota-rod test)	Delayed righting reflex and eye opening, decreased cliff avoidance, no difference in motor coordination
Abu-Taweel 2012 [30]	Mice/SW	15 or 30 mg/kg/day in water	Distilled water	G1 until PND 15	22 days	Eye opening. Righting reflex, cliff avoidance, rotating reflex, locomotor activity test	Delayed eye opening. Inhibitory dose-dependent effect on sensory motor reflexes and locomotor activity
Nery (2014) [31]	Zebrafish/Daniorerio	0.05 mM, 0.5 mM, 5 mM	System water	G3	10 days	Locomotor activity	Dose-dependent locomotor deficit

*s.c.* subcutaneous, *n.r.* not reported, *G* gestation day, *PND* postnatal day, *SW* Swiss Webster, *SD* Sprague–Dawley, *W* Wistar

**Table 2:** Characteristics and results of preclinical studies on intrauterine exposure to antipsychotics

Author (year)	Species/strain	Experimental medication (dosage)	Control medication	Exposed period	Follow-up time	Measurements	Results
Jewett (1966) [59]	Rats/SD	Cpz 2 mg/ml or Inj 0.1 ml/100 g body weight 3× daily	Distilled water Inj 3× daily or no treatment	G4–G7	75 days	Spontaneous motor activity (photoelectric cell activity cage), audiogenic seizures	Cpz: decreased motor activity day 30–33; no difference day 23–26. Increased susceptibility to audiogenic seizures
Ordy (1966) [63]	Mice/C57BL/10	Cpz 4 or 16 mg/kg/day orally	Placebo	G6–PND0	60 days	Open field test, wheel running activity, shock-elicited escape avoidance conditioning	Delayed open field latency to move to middle square. Fewer rotations in wheel running. Fewer avoidances in conditioning
Hoffeld (1968) [58]	Rats/SD	Cpz 6.0 mg/kg/day	Distilled water	G5–G8 (I) G11–G14 (II) G17–G20(III)	97 days	Rotary activity wheel, emotionality testing (faecal boluses), stress reaction (stomach ulcers)	Increased activity. More activity in 2nd and 3rd than in 1st trimester exposed pups. No difference in emotionality and stress response
Clark (1970) [61]	Rats/SD	Cpz 3 mg/kg/day s.c.	Vehicle	G12–G15	60 days	Open field test, T-maze, mother-goal maze, operant conditioning	Locomotor activity reduced on day 13 but enhanced on day 18. Maze learning: shorter latencies, higher error scores in mother-goal maze; no differences in T-maze. Operant conditioning: one more session needed to acquire bar-press response
Robertson (1979) [60]	Rats/CR	Cpz 1, 3 or 9 mg/kg/day by gavage	Vehicle	G6–G 15	13 weeks	Open field test in week 3,7,13, brain weight in week 15	Increased open field activity and decreased latency time in week 3 and 13 in the 3 and 9 mg group. No difference in brain weight
Spear (1980) [50]	Rats/SD	Hal 0.25 mg/kg/cc in water	Distilled water	G1–PND21	54 days	Open field test, open field hole-poking, response to amphetamine and haloperidol	Increased locomotor activity, hole-poking and response to amphetamine. Accentuated response to haloperidol in early life and young adulthood but not in adolescence
Umemura (1983) [62]	Rats/SD	Cpz 2 or 8 mg/kg/day s.c.	Saline	G17–PND21	15 weeks	Spontaneous motor activity (magnatic field activity counter) and light–dark discrimination learning test	No difference in spontaneous activity. Impairment of reversal learning
Hull (1984) [52]	Rats/LE	Hal 2.5 mg/kg/d i.p.	Saline i.p. (4 groups with Hal and saline pre- and postnatally)	G7–PND21	79 days	Open field test, eye opening, haloperidol-induced catalepsy	No difference in open field ambulation, eye opening or haloperidol-induced catalepsy
Szkilnik (1987) [64]	Rats/W	Cpz 1 or 5 mg/kg/d s.c.	Saline 1 ml/kg/day s.c.	G1–PND21	3 months	Lats’ test, open field test, hole test, chlorpromazine-induced catalepsy, conditional avoidance learning	No difference in Lats’ test. Lower number of trespassings and lookings outside. Increased excitability. Fewer dippings in hole test. Increased catalepsy. No difference in conditional avoidance learning
Bruses (1989) [45]	Rats/SD	Hal 2.5 mg/kg/day i.p.	Saline 200 µl i.p.	G5–G20	38 days	Surface righting reflex, negative stereotaxis test, T-maze spatial learning, circling training	Delayed surface righting reflex, fewer turns on circling training, no difference in T-maze spatial learning test
Scalzo (1989) [55]	Rats/SD	Hal 2,5 or 5 mg/kg/day s.c.	Vehicle	G6–G20	62 days	Milk induced behavioural activation (day 6), SPWC (day 9,11,13,15,17), stimulant induced behavioral stereotypes (SIBS) (day 30), duration of barbiturate anesthesia (day 34, 62)	SPWC duration reduced on day 9 + 11 but not later. Reduced total anesthesia duration at day 62 in 5 mg group. No difference in milk induced behavior or SIBS
Myslivecek (1991) [57]	Rats/W	Cpz 2.5 mg/kg/day Inj	Saline Inj	G15, 18, 20	4 months	Eye opening, righting reflexes, hanging, passive avoidance learning paradigms (neonatal, 2 months, 4 months)	No difference in eye opening. Delayed righting reflexes. Impaired hanging. Impaired passive avoidance learning
Archer (1992) [47]	Rats/n.r.	Hal 2.5 µmol/kg/day by gavage	Vehicle	G6–G21	25 days	Radial arm maze and circular swim maze. Response to low dose d-amphetamine	Increased locomotion, rearing, and total activity. Rearing behavior reduced 90 min after d-amphetamine, potentiation after 120 min. Potentiation of stimulatory effect of d-amphetamine on locomotion. Retardation of spatial learning.
Williams (1992) [56]	Rats/SD	Hal 5 mg/kg/d s.c.	Vehicle	G6–G20	100 days	Brain weight	Decreased brain weight
Singh (1997) [49]	Rats/CF	Hal 0.1 mg/kg/day i.p.	Vehicle	G13–G20	7–8 weeks	Open field test, tunnel-entry test, elevated zero maze test, elevated plus-maze test	Increased ambulation and rearing. Decreased scratching, licking and washing behavior in open field. Tunnel: decreased time in centre of cage. Zero-maze: less time in open arms. Plus maze: fewer entries and less time in open arms
Singh (1998) [54]	Rats/n.r.	Hal 0.1 mg/kg/d i.p.	Vehicle	G13–G21	8 weeks	Foot shock induced aggressive behaviour test	Increased number of fighting bouts. No difference in fighting latency
Rosengarten (2002) [48]	Rats/SD	Hal 2 mg Qtp 10 mg Olz 2 mg Ris 1 mg/kg/day in water	Vehicle	G8–G18	2 months	Radial arm maze: spatial learning and short term retention	Hal,Ris,Qtp: impaired spatial learning, Hal, Ris: impaired short-term retention, Olz: no differences in spatial learning or short-term retention
Singh (2002) [51]	Rats/CF	Hal 2.5 mg/kg/day i.p.	Vehicle	G12–G20	56 days	Open field test, elevated plus-maze test, zero-maze test (anxiety patterns)	Increased ambulation, rearing and defecation. Plus-maze: fewer entries and less time in open arms, more entries and time in closed arms. Zero-maze: fewer head dips and stretch attend postures
Wolansky (2004) [46]	Rats/SD	Hal 2.5 mg/kg i.p.	Vehicle	G5–G18	90 days	Circling training test	Decreased circling activity, but effect disappeared when exposure was continued during lactation
Zuo (2008) [68]	Rats/SD	Ris 2 mg/kg, Sul 200 mg/kg in water	Saline in drinking water	G6–G18	60 days	Righting reflex, Open field test, Morris water maze	Ris: increased rearing. No difference in water maze tests or righting reflex. Sul: impaired cue response in visual task performance (Morris water maze). Reduction in spontaneous activity. No difference in righting reflex
Singh (2015) [66]	Rats/W	Qtp 55 or 80 mg/kg/day orally	Vehicle	G6–G21	70 days	Morris water maze; passive avoidance learning task	Impaired (dose-dependent) spatial learning. Impaired retention capability
Singh (2016) [67]	Rats/W	Ris 0.8 mg/kg/day; 1.0 mg/kg/day; 2.0 mg/kg/day in water	Saline	G6–G21	10 weeks	Open field test, elevated plus-maze, brain weight	Increased ambulation and rearing. Anxiety-like exploratory behavior. Dose-dependent reduction in brain weight

*SD* Sprague–Dawley, *W* Wistar, *CF* Charles–Foster, *LE* Long–Evans, *CR* Charles–River, *n.r.* not reported, *Cpz* Chlorpromazine, *Hal* haloperidol, *Ris* Risperidone, *Qtp* Quetiapine, *Sul* Sulpiride, *Olz* Olanzapine, *SIBS* stimulant induced behavioral stereotypes, *s.c.* subcutaneous, *i.p.* intraperitoneal, *G* gestation day, *PND* postnatal day, *Inj* injection, *SPWC* shock precipitated wall climbing

**Table 3:** Characteristics of clinical studies on neurodevelopmental outcome after intrauterine exposure to lithium

Author (year)	Study design	Sample size	Lithium daily dosage	Treatment indication	Follow-up time	Measurements	Results	NOS
Schou (1976) [36]	Prospective cohort study	Exposed = 60 Controls = 57	n.r.	n.r.	Mean: 7 years	Developmental questionnaire^a^	No difference in rate of abnormal development	7
Jacobson (1992) [37]	Prospective cohort study	Exposed = 22 Controls = n.r.	Mean: 927 mg	Major affective disorders	Mean: 61 weeks, range: 1-9 years	Telephone interview on the attainment of developmental milestones	No difference	3
vd Lugt (2012) [38]	Cohort study	Exposed = 15 No controls	n.r.	Bipolar disorder	3–15 years	Development questionnaire^a^ IQ by BSID or WPPSI/WISC Hempel or Touwen neurological examination Child Behavior Checklist*	MND (*n* = 1). Low V–IQ + T–IQ normal P–IQ (*n* = 1) Subclinical anxiety problems (*n* = 2). Subclinical oppositional problems (*n* = 1)	6

*n.r.* not reported, *BSID* Bayley Scale of Infant Development, *WPPSI* Wechsler Preschool and Primary Scale of Intelligence, *WISC* Wechsler Intelligence Scale for Children, *MND* minor neurologic dysfunction; NOS: Newcastle–Ottawa Scale

^a^parent report

**Table 4:** Characteristics of clinical studies on neurodevelopmental outcome after intrauterine exposure to antipsychotics

Author (year)	Study design	Sample size	Medication (daily dosage)	Treatment indication	Follow-up time	Measurements	Results	NOS
Slone (1977) [77]	Prospective cohort study	Exposed = 2141 Controls = 26.217	Phenothiazine antipsychotics (n.r.)	n.r.	4 years	IQ scores	No difference	5
Platt (1989) [73]	Prospective cohort study	Exposed = 192 D+ Med− = 116	Antipsychotic neuroleptics (n.r.)	Psychotic neurotic disorders	7 years	Motor development in newborn period, at 8 months, 4 and 7 years	Newborn: increased abnormal motor activity; 8 months: trend towards more failures on BSID (not sign.)	6
Stika (1990) [76]	Cohort study	Exposed = 66 Controls = 66	Chlorpromazine (10–25 mg) or chlorprotixene (5 mg)	n.r.	10 years	Teacher questionnaire	No difference in behavioural score	5
Auerbach (1992) [69]	Cohort study	Exposed = 14 Controls = 26 D+ Med − = 18	Phenothiazine antipsychotics (varied)	SMI	14 days	NBAS at day 3 and day 14	Reduced autonomic stability and higher abstinence score.	8
Mortensen (2003) [71]	Register study	Exposed = 63 Controls = 755	Neuroleptics (n.r.)	n.r.	7–10 months	Boel test	Adjusted OR abnormal Boel test in exposed children = 4.1 (95% CI 1.3–13.0)	7
Johnson (2012) [70]	Prospective cohort study	Exposed = 21 Controls = 78 ADD exposed = 183	Antipsychotics combined (n.r.)	Anxiety and Mood Disorders	6 months	INFANIB	Lower INFANIB scores after exposure to antipsychotics	7
Peng (2013) [72]	Prospective cohort study	Exposed = 76 Controls = 76	33 Clz (178 mg), 16 Ris (2 mg), 13 Sul (461 mg), 8 Olz (8 mg), 6 Qtp (550 mg)	Schizophrenia	12 months	BSID at 2, 6, 12 months	2 months: lower on cognitive, motor, social-emotional and adaptive behavior scale, 6 months: lower on social-emotional and adaptive behavior scale, 12 months: no difference	7
Shao (2015)^a^ [80]	Post hoc analysis (Peng 2013)	Exposed = 63	33 Clz (178 mg), 30 other AP (16 Ris (2 mg), 8 Olz (8 mg), 6 Qtp (550 mg))	Schizophrenia	12 months	BSID at 2, 6, 12 months	2 and 6 months: lower adaptive behavior scores for Clz exposed children compared to other AP, 12 months: no difference	
Hurault-Delarue (2016) [74]	Register study	Exposed = 70 Controls = 32.303	Neuroleptics (n.r.)	n.r.	24 months	Pediatric examination	9 months: higher prevalence of motor deficits, no difference in mental development, 24 months: no difference	7
Petersen (2016) [75]	Register study	Exposed = 290 Controls = 210.966 D+ Med− = 492	Antipsychotics (n.r.)	SMI	9 months to 5 years	NDBD reported in health record	No difference in relative risk of NDBD after adjustment for confounders [RRR 1.22 (95% CI 0.80–1.84)]	8

*n.r.* not reported; *D+ Med−* control group of women with comparable treatment indication but no medication use during pregnancy, *SMI* severe mental illness, *NDBD* neurodevelopment disorders and behavioural disorders, *BSID* Bayley Scale of Infant and Toddler Development, *ADD* antidepressant drugs, *INFANIB* infant neurological international battery, *NBAS* Neonatal Brazelton Assessment Scale, *Clz* clozapine, *Ris* risperidone, *Sul* sulpride, *Qtp* quetiapine, *AP* antipsychotics, *OR* odds ratio, *RRR* relative risk ratio, *NOS* Newcastle–Ottawa scale

^a^post hoc analysis on subsample of cohort study by Peng 2013

Of the 73 studies included in the qualitative analysis, 29 were preclinical investigations of which seven examined lithium exposure and 22 examined antipsychotics exposure. Most preclinical studies were performed in rats, some in mice and one study on lithium exposure used zebrafish. There is a large variety of the measurements used to assess neurodevelopment in animal models (Table 1, 2). The exposed period was generally during gestation, although several studies also investigated the effect of exposure during lactation. Postnatal brain development in rodents up to postnatal day 10 is considered analogous to prenatal brain development in humans [28].

In total, we found 13 clinical cohort studies of which three involved lithium exposure and ten involved antipsychotics exposure (Table 3, 4). Study samples varied from 14 to 2141 exposed subjects. Mean follow-up duration ranged from 1 to 15 years in studies involving lithium exposure and from 14 days to 5 years in studies involving antipsychotic exposure. Assessment of neurodevelopment varied between cohort studies. Out of the three clinical studies involving lithium exposure, one used standardized assessments, while the other two relied on an invalidated questionnaire or telephone interview. Most studies involving antipsychotic exposure used standardized objective assessments, but some studies relied solely on invalidated questionnaires or interview. Additionally, 31 case studies were included, of which 5 involved intrauterine lithium exposure and 26 involved intrauterine antipsychotics exposure (Supplement 2).

## Lithium

### Preclinical investigations

Sechzer et al. [29] investigated the long-term developmental consequences of prenatal and early postnatal lithium exposure in rats. Female rats were treated with lithium during pregnancy and lactation. Development of the startle response and depth perception in the offspring were delayed. At the age of 4 months, pups exhibited lower spontaneous activity during open field activity testing. A similar study investigated the neurodevelopmental effect of lithium exposure from day 1 of pregnancy until postnatal day 15 [30]. Decreased locomotor activity and delayed development of sensory motor reflexes were observed in lithium-exposed mice. Whether these developmental delays were caused by prenatal or early postnatal exposure to lithium could not be determined. Nery et al. [31] studied the behavioural effects of lithium exposure on the development of zebrafish embryos and reported decreased locomotion compared to non-exposed embryos. Additionally, several studies have replicated a delay in eye opening [29, 30, 32] and decreased avoidance behaviour [32, 33] in mice and rats exposed to lithium during gestation and/or lactation. One study found impaired performance on the T-maze test [33]. Messiha et al. [34] found lower brain weights in lithium exposed offspring at the age of 37 days. No changes in social, defensive, threatening or aggressive behaviour was observed in lithium-exposed mice [35].

In summary, preclinical studies suggest a deleterious effect of lithium on motor activity, developmental milestones and reflexes, spatial memory and brain weight.

### Clinical investigations

Neurodevelopment of 97 children with in utero exposure to lithium has been investigated in clinical cohort studies. Overall, most children were reported to have typical neurodevelopmental trajectories. Schou analysed data from the Scandinavian Register of Lithium Babies to compare neurodevelopmental outcomes in lithium-exposed children (*n* = 60) with their non-exposed siblings (*n* = 57) (average age, 7 years) [36]. Outcomes were assessed by questionnaire and based solely on mothers’ subjective retrospective assessment of their children’s developmental milestones. No significant differences were observed between the lithium-exposed children and their siblings.

In a prospective multicenter study, major developmental milestones were examined between a sample of 22 lithium-exposed children with non-exposed children [37]. Subjects were screened for study inclusion from among mothers who contacted the public teratogen information services to discuss the potential risks of prescription medication use during pregnancy. Data were collected by telephone interview. No differences were observed between lithium-exposed versus non-exposed children in the age at which they achieved major developmental milestones.

In an observational cohort study, 15 lithium-exposed children between 3 and 15 years old were investigated [38]. Standardized validated tests were used to assess growth, neurological, cognitive and behavioural outcomes. When compared to norms from the general population, most lithium-exposed children scored lower on the Block patterns subtest of the Wechsler Intelligence Scale for Children (WISC-III-NL). In contrast, no differences in growth or behavioural outcomes were observed. One child in this study exhibited subclinical neurological findings. Importantly, however, the conclusiveness of this study was hampered by the lack of a matched non-exposed control group ascertained in parallel with the lithium-exposed group, but rather relied upon an independently collected general population cohort dataset.

In summary, there is a paucity of clinical data on the neurodevelopment of children with in utero exposure to lithium. The three clinical studies published in the literature report normal neurodevelopment.

### Case reports

Neurodevelopmental delay after intrauterine exposure to lithium was reported in four case studies, encompassing a total of eight children [39–42]. Kozma et al. [39] reported on a neonate with neurodevelopmental deficits, including decreased muscle tone, depressed reflexes and diminished social response, during the 2.5 months after birth. However, by 13 months of age, no deficits were observed using the Bayley Scale of Infant Development. Morrel et al. [43] described a case of lithium toxicity at 35 weeks of gestation (lithium blood level: 2.6 mmol/L). The baby was born with primary cardiac muscle dysfunction and treated with isoprenaline at birth. At 12 months of age, cardiac function had normalized but there was evidence of delayed motor development and a concomitant strabismus. Delayed gross motor function was also reported in two cases with prenatal lithium exposure that did not involve lithium intoxication [41, 42]. One case report reported normal psychomotor development [44].

## Antipsychotics

### Preclinical investigations

Eleven studies have investigated the long-term neurodevelopmental consequences of prenatal haloperidol exposure in rats. Emergence of the surface righting reflex was found to be delayed [45]. Two studies found deficits in a circling training test [45, 46], a measure of motor performance and associative learning. Impairments in spatial learning were also found [45, 47, 48] using the Morris water maze, T-maze and radial arm maze. Moreover, the open field test revealed increased rearing, ambulation and general activity [47, 49–51]. One study reported finding no difference in ambulation on the open field test [52]. Notably, since behaviour in the open field test is influenced not only by locomotor activity, but also by anxiety and exploratory behaviour [53], additional studies have been performed to further differentiate these phenotypes. Indeed, consistent with an increase in anxiety, rats made fewer entries and spent less time in open arms during elevated zero and plus-maze tests [49, 51]. Moreover, aggressive behaviour was also increased [54]. Duration of shock-precipitated wall climbing was reduced on postnatal days 9 and 11, and there were no differences in stimulant induced behavioural stereotypes [55]. Lastly, from a neuroanatomical perspective, rats with intrauterine exposure to haloperidol exhibited significantly lower brain weight in adulthood [56].

Eight studies have investigated the long-term neurodevelopmental effects of intrauterine exposure to chlorpromazine in rats. One study systematically investigated the onset of neurodevelopmental milestones [57]. They found no difference in onset of eye opening, but emergence of the righting reflex was delayed. Studies investigating motor development found both increases [58] and decreases [59] in wheel running activity and impairments in a hanging task [57]. In the open field test, latency time was decreased [59, 60] and locomotor activity was increased [60, 61]. Similarly, spontaneous activity was normal in one study [62], but decreased in another [63]. Spatial memory was found to be impaired in a mother-goal maze task, whereas no differences were found in a T-maze task [61]. Studies focusing on other types of learning have reported impaired avoidance conditioning [57, 59], reversal learning [62] and operant conditioning [61]. Avoidance conditioning was observed to be normal [64]. A study investigating exploratory behaviour found that rats made fewer hole dippings [64]. Hoffeld et al. [58] did not find changes in emotionality testing. Susceptibility to audiogenic seizures was increased [63]. Brain weights did not differ between chlorpromazine and placebo exposed groups [60].

Several other antipsychotics were examined for neurodevelopmental effects. Rosengarten et al. [65] investigated the possible sequelae of intrauterine exposure to quetiapine, risperidone or olanzapine and found impaired spatial learning in a radial arm maze task for both risperidone and quetiapine, and disrupted short-term retention for quetiapine. Intrauterine exposure to olanzapine did not affect learning or retention. A recently published study also found impaired spatial learning and retention capability in rats with prenatal exposure to quetiapine [66]. Two studies investigated the effects of risperidone exposure during gestation. Singh et al. [67] reported increased ambulation and rearing in the open field test, and increased anxiety-like exploratory behavior in the elevated plus maze test. Intrauterine exposure to risperidone led to a dose-dependent reduction of adult brain weight. Zuo et al. [68] also found increased ambulation, while righting reflexes and spatial memory were normal. In the same study, rats with prenatal exposure to sulpiride exhibited an impaired cue response in a visual task performance and reduced spontaneous activity, while righting reflexes and spatial memory were normal.

In summary, most preclinical studies found a deleterious effect of antipsychotics on motor activity, developmental milestones and reflexes and spatial memory. Additionally, exposure to either haloperidol or risperidone led to decreased brain weight.

### Clinical investigations

In total, neurodevelopmental data of 2934 children with in utero exposure to antipsychotics have been published involving nine clinical cohort studies. Six studies reported neurodevelopmental delays or deficits after prenatal exposure to antipsychotics [69–74], while three studies reported normal developmental outcomes [75–77]. Most studies reported antipsychotic exposure on the basis of a single broad category, which combined a wide variety of antipsychotics. The initial report of abnormalities of motor development in children with intrauterine exposure to antipsychotic was authored by Platt in a cohort of 192 children exposed to antipsychotic neuroleptics and 116 children of women with a history of psychiatric disorders described as psychotic/neurotic but without antipsychotic treatment. Notably, deficits at the neonatal assessment of motor activity were more severe than at 8 months of age, when there was a non-significant trend towards more failures based on the Bayley gross motor assessment [73]. Another study examined 21 children with prenatal antipsychotic exposure, 183 children with prenatal antidepressant exposure and 78 non-exposed children at 6 months of age [78]. Children with prenatal exposure to antipsychotics had lower scores on the infant neurological international battery (INFANIB) compared to children with prenatal antidepressant exposure or non-exposed children. Comparable results were found in a recent register-based study in France [74]. Psychomotor development, assessed by pediatric examination, was compared between 70 children with prenatal neuroleptic exposure and 32.303 non-exposed controls. A higher prevalence of motor deficits was reported in exposed children at 9 months of age, a difference that was no longer present at 24 months of age. No differences in cognitive development were observed. A Danish general population register-based study reported an association between drug prescriptions during pregnancy and results on the Boel test, a psychomotor development test assessed at 7–10 months of age [71]. Specifically, the odds ratio for an abnormal Boel test was 4.1 (95% CI 1.3–13.0) among children with intrauterine exposure to neuroleptic medication after adjustment for several confounders including gestational age, birth weight and breastfeeding. In contrast, Stika et al. [76] reported finding no discernible adverse behavioural outcomes based on evaluations made by their classroom teachers in 10-year-old children with prenatal neuroleptic exposure.

Using data from two large electronic primary care databases in the UK, Petersen et al. investigated the risks and benefits of psychotropic medication during pregnancy. They compared the prevalence of neurodevelopmental and behavioural disorders in children with prenatal exposure to antipsychotics, children with no antipsychotic exposure whose mothers discontinued antipsychotic treatment before pregnancy and non-exposed children whose mother was not prescribed antipsychotic treatment in the 24 months before pregnancy. They found an increased risk of neurodevelopmental and behavioural disorders in children exposed to antipsychotics with a relative risk ratio (RRR) of 1.58 (95% CI 1.04–2.40). However, after adjustment for possible confounders, these differences were no longer statistically significant (RRR 1.22, 95% CI 0.80–1.84) [75]. An earlier study, focused specifically on phenothiazine antipsychotics, similarly reported no difference in intelligence quotient scores among 4-year-old children, of which 2141 had prenatal exposure and 26,217 were non-exposed [77]. Notably, however, a higher burden of neonatal withdrawal symptoms and autonomic instability was reported 14 days after birth in neonates with intrauterine exposure to phenothiazine antipsychotics [69].

More recent studies have also focused on the long-term developmental consequences of intrauterine exposure to atypical antipsychotics. Peng et al. [79] prospectively investigated 76 children with intrauterine exposure to atypical antipsychotics and 76 non-exposed controls, from birth until 12 months of age. Neurobehavioural development was assessed by the Bayley Scale of Infant and Toddler Development (BSID) at 2, 6 and 12 months of age. At 2 months of age, antipsychotic-exposed children exhibited significantly lower scores regarding cognitive, motor, social-emotional, and adaptive behavioural functioning. At 6 months of age, scores regarding social-emotional and adaptive behavioural functioning were still lower, but not significantly different between groups in cognitive or motor scores. In contrast, by 12 months of age none of these effects persisted. A post hoc analysis revealed that children prenatally exposed to clozapine had lower scores on the BSID adaptive behavior scale at the ages of 2 and 6 months compared to children exposed to other atypical antipsychotics [80]. However, this difference was also no longer present at 12 months of age.

Although studies varied in measurements and follow-up time, five cohort studies [70–74, 80] investigated motor development of children with in utero exposure to antipsychotics. These studies consistently showed a deficit in motor functioning in the first 9 months of life, but which appeared to spontaneously resolve based on subsequent follow-up assessments.

### Case reports

Overall, case studies on intrauterine exposure to first generation antipsychotics have largely reported normal neurodevelopment [81–88]. Additionally, although most case studies involving prenatal exposure to second generation antipsychotics have also reported normal infant and child development [81, 88–101], several case reports have found neurodevelopmental delays or deficits. Two cases reported speech delay, one involving risperidone and the other clozapine [102, 103]. One case reported abnormal behavioral development following prenatal exposure to risperidone and ziprasidone [103]. Impaired motor development has been reported following exposure to olanzapine, clozapine or risperidone [104–106].

### Risk of bias and quality of the included studies

The risk of bias for the included preclinical studies is presented in the Supplementary Material (Supplement 3). Notably, many lack descriptions of the assessed domains, thereby making the risk of bias unclear (e.g., selection bias, performance bias, detection bias or attrition bias). In 34% of the preclinical studies, cross-fostering after birth was applied in order to control for medication-induced changes in maternal care.

NOS scores of clinical cohort studies varied between three and eight points (Table 3 and 4). Only three cohort studies properly controlled for maternal mental illness, widely considered the most important confounder in studies of intrauterine exposure to prescription psychotropic medication. In the other studies, neurodevelopment was compared between children with prenatal exposure to lithium or antipsychotics versus unaffected children, thereby leaving unaddressed the risk of confounding by indication. Moreover, few clinical studies controlled for additional confounders such as maternal age, congenital malformations, preterm birth, or smoking and alcohol use during pregnancy, often because information on these factors was not available. In most studies of antipsychotic exposure, developmental assessments were standardized and validated, although some studies based their results on non-validated questionnaires or information obtained exclusively from medical records. The quality of the included studies on lithium exposure is poor, as only one cohort study used validated measurements of neurodevelopment. Unfortunately, this study did not compare their findings with a formal control group. Regarding case studies, their quality is generally considered low with a high risk of publication bias. Indeed, most case studies did not assess neurodevelopment using validated objective measures.

### Meta-analysis

**Fig. 2:**
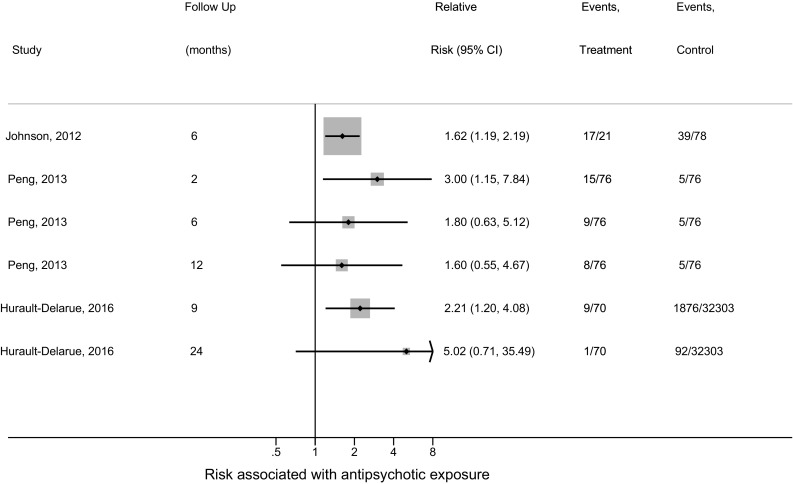
Relative risk estimates including the 95% confidence interval limits of neuromotor deficits for antipsychotic exposure for all reported follow-up assessments

Three out of five studies that investigated neuromotor deficits in children with in utero exposure to antipsychotics provided sufficient data and were included in a meta-analysis [70, 72, 74]. Figure 2 shows the relative risk of neuromotor deficits for antipsychotic exposure for all reported follow-up assessments (six effect sizes). Pooled relative risk calculated using fixed effect estimation was 1.97 (95%CI 1.47–2.62; *Z* value: 4.59, *p* < 0.001) with absence of heterogeneity (*I*^2^ 0%, *p* = 0.622). Since studies reported multiple follow-up outcomes, this pooled estimate should be interpreted with care.

**Fig. 3:**
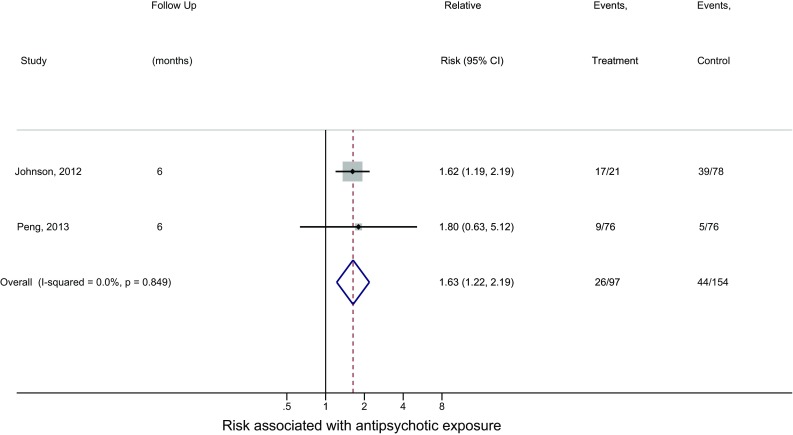
Relative risk estimates including the 95% confidence interval of neuromotor deficits for antipsychotic exposure at 6 months of follow-up. The pooled relative risk was estimated using a fixed-effects estimation

Two studies [70, 72] reported follow-up outcomes at 6 months. The pooled relative risk was 1.63 (95% CI 1.22–2.19; *Z* value = 3.29; *p* = 0.001, fixed effect) with absence of heterogeneity (*I*^2^ = 0%, *p* = 0.849), indicating a 63% increased risk for neuromotor deficits at 6 months (Fig. 3).

Next we performed a random effects meta-regression analysis to study the longitudinal influence of intrauterine exposure to antipsychotics on motor development. For each study, we included the initial follow-up assessment. The direction of the regression coefficient suggested a decrease of the impact of intrauterine exposure to antipsychotics on neuromotor deficits over time. However, there was no statistically significant effect (− 0.03; 95% CI − 1.26 to 1.20; *p* = 0.80). Residual heterogeneity was substantial (*I*^2^ = 49%). In conclusion, in this meta-analysis we were able to partially confirm the negative effect of antipsychotic exposure on motor development. However, we were not able to confirm the transient nature of the neuromotor deficits.

## Discussion

In this systematic review article and meta-analysis, we present an overview of the current literature regarding long-term neurodevelopmental effects of lithium and antipsychotics. Towards this goal, we included both preclinical and clinical studies. Preclinical studies have the potential to investigate the effect of medication exposure using more optimal study designs in which important biases in clinical studies, such as confounding by indication, can be directly addressed. Notably, although preclinical findings may not always be translatable into clinical practice, they have the potential to provide mechanistic insights and reveal indications of possible risks in situations for which well-controlled high-quality clinical studies are lacking. Undoubtedly, however, disproportionate weight should be given to evidence discerned from relevant clinical studies when helping women to consider the risks and benefits of their perinatal treatment options.

Overall, findings from preclinical studies suggest a deleterious effect of lithium on locomotor activity and delayed development of eye opening and righting reflexes. Additionally, brain weight was found to be lower in lithium-exposed offspring. Clinical studies of offspring neurodevelopment after intrauterine exposure to lithium generally reported normal development. However, two out of the three studies based their results exclusively on retrospective maternal reports, while the third study lacked a formal control group. The lack of clinical studies on the risks of lithium use during pregnancy might be due to the fact that lithium is a naturally occurring element that was never patented. Another explanation for the knowledge gap on long-term neurodevelopmental effects of lithium exposure might be the (earlier recognized) association with cardiac malformations. This association was first reported in the 1970′s by Schou et al. and a recent study by Patorno et al. confirmed this association although the authors report that the risk was lower than previously suggested [10, 107]. These findings have influenced treatment guidelines in the United Stated and the United Kingdom, where lithium use during pregnancy is discouraged [108, 109], and possibly also influenced research to focus on congenital malformations rather than on neurodevelopment.

Despite the many differences in methodology, preclinical studies consistently reported adverse neurodevelopmental and behavioural effects of prenatal exposure to antipsychotics. Antipsychotics seem to increase locomotor activity and anxiety, as well as impair cognition, in exposed offspring. Lastly, and of important consideration for clinical translational potential, brain weight was found to be lower in offspring with intrauterine exposure to haloperidol and risperidone. Most studies of antipsychotics involved haloperidol and chlorpromazine, while a much smaller number focused on varied atypical antipsychotics. At present, there is insufficient evidence to conclude whether the neurodevelopmental impact of prenatal exposure to antipsychotics is dependent upon the specific type or class. Findings from clinical studies of antipsychotic exposure are inconsistent and difficult to interpret due to the considerable differences in methodology and follow-up period. Several studies reported a delay in neurodevelopment among infants with intrauterine exposure to antipsychotics. However, these early neurodevelopmental delays were frequently transient, having resolved on subsequent longitudinal follow-up assessments. The most consistent finding was a transient delay in motor development. This was confirmed in our meta-analysis with a relative risk of 1.36 for neuromotor deficits after in utero exposure to antipsychotics at 6 months of follow-up. However, this estimate was based on only two studies. More studies are needed to provide a more robust estimate and to study the course of motor development over time. Most studies had a follow-up period of less than 2 years, for which later-onset neurodevelopmental sequelae cannot be excluded. Based on the currently available reports, no distinction between the various types of antipsychotics can be made as most studies combined different types and classes of antipsychotics into a single broad category, presumably to increase statistical power.

Clinical findings might have been affected by confounding by indication, since most studies compared exposed children to non-exposed children of mothers with no history of psychiatric illness. Therefore, studies have not been able to adequately adjust for genetic predisposition, psychiatric illness during pregnancy, or parenting, all of which would be expected to independently influence child development [110–112]. Regardless of medication exposure, offspring of patients with schizophrenia and bipolar disorder have an increased risk to develop any mental illness [113] and experience more cognitive impairments [114–116]. A recent study found impairment of motor function among children with a familial risk of schizophrenia [117]. Additionally, studies using structural MRI have reported decreased white and gray matter volume in offspring of parents with bipolar disorder or schizophrenia [118–121]. It is therefore of particular importance for future studies to compare psychotropic medication-exposed children to non-exposed children of mothers with similar psychopathology.

Our findings may have been influenced by publication bias, since studies without significant results are less likely to be published [122]. This is particularly the case for preclinical studies. As a result, the rate and severity of neurodevelopmental deficits presented in this review might be an overestimation. However, the paucity of evidence regarding the long-term effects of intrauterine exposure to lithium or antipsychotics may also lead to a blunted motivation to invest in studies of the potential adverse neurodevelopmental consequences and consequent underreporting of associations. Undoubtedly more studies of higher quality will be required in order to address these questions with greater certainty.

Our results show a discrepancy between findings from preclinical and clinical studies, with preclinical studies reporting more discernible neurodevelopmental deficits. As mentioned above, publication bias might be part of the explanation. In addition, many preclinical studies used high dosages of medications, exceeding 80% occupancy of the D2-receptor causing more side-effects [123]. Lastly, species differences cannot be disregarded as a potential source of discrepancy between pharmacological studies in animals and humans.

High quality clinical studies will be required in order to properly assess the risk of adverse neurodevelopmental effects of intrauterine exposure to lithium and antipsychotics. Randomized controlled trials are often considered the best approach to studying causal inference. However, there is broad consensus that randomized assignment for the purpose of studying medication side effects is unethical [124]. Furthermore, placebo-controlled randomization of women with mental health indications for lithium or antipsychotics is also considered unethical when treatment is medically indicated, but also regarding exposure of the fetus when treatment is not medically indicated. Future studies of neurodevelopmental outcome in children with intrauterine exposure to psychotropic medication will therefore have to continue to rely upon clinical cohort studies, for which non-randomized designs can be well suited for studying unintended pharmacological effects [125]. However, cohort studies should ideally have a prospective design with extended follow-up periods utilizing validated standardized neurodevelopmental outcome measures. Moreover, in an effort to reduce confounding by indication, the primary comparison group for exposed children should involve non-exposed children of mothers with similar psychopathology. Since it is unlikely that medicated and non-medicated pregnant women have the same disease severity, cohort studies should also consider designs in which pregnant women treated with lithium or antipsychotics are compared to pregnant women with the same psychiatric disorder but other pharmacological treatments.

The decision for pharmacological treatment during pregnancy should always be decided through a patient-centered discussion with their healthcare provider by carefully weighing the risks and benefits of various treatment options and by developing an individualised treatment plan.

## Conclusion

Prenatal exposure to lithium or antipsychotics has an adverse effect on neurodevelopment and behaviour in mice and rats, but the precise mechanisms remain unclear. In humans, the existence and nature of any effects remains poorly determined. At present, there is insufficient evidence to estimate the neurodevelopmental effects of intrauterine exposure to lithium. Although several studies have reported a transient neurodevelopmental delay following intrauterine exposure to antipsychotics, the current lack of high quality clinical investigations substantially limits the conclusiveness of the available evidence. In particular, improved clinical studies will require prospective designs with longer follow-up periods and more extensive assessments including validated measures of child development, in order to offer more substantiated evidence-based advice to women with bipolar disorder or psychotic disorders regarding the risks and benefits of pharmacotherapy during pregnancy.

## Electronic supplementary material

Below is the link to the electronic supplementary material.

Supplementary material 1 (DOCX 20 kb)

Supplementary material 2 (DOCX 23 kb)

Supplementary material 3 (PDF 41 kb)
