# Supplementary material for: Long-term neurodevelopmental consequences of intrauterine exposure to lithium and antipsychotics: a systematic review and meta-analysis
Source: Eur Child Adolesc Psychiatry. 2018 Jun 11;27(9):1209–30. doi: 10.1007/s00787-018-1177-1 (PMC6133089; doi:10.1007/s00787-018-1177-1)
Supplement: Supplementary file 1 — Supplementary material 1 (DOCX 20 kb) [file 787_2018_1177_MOESM1_ESM.docx]

**Long-term neurodevelopmental consequences of intrauterine exposure to lithium and antipsychotics: a systematic review and meta-analysis**

Eline M. P. Poels^1^, Lisanne Schrijver^1^, Astrid M. Kamperman^1^, Manon H.J. Hillegers^2^, Witte J. G. Hoogendijk^1^, Steven A. Kushner^1^, Sabine J. Roza^1^

^1^ Department of Psychiatry, Erasmus University Medical Center, ’s-Gravendijkwal 230, 3015 CE, Rotterdam, The Netherlands

^2^ Department of Child and Adolescent Psychiatry, Erasmus University Medical Center, Rotterdam, The Netherlands

**Supplementary material 1**

Search terms per database:

**Embase.com**

(lithium/exp OR 'lithium acetate'/exp OR 'lithium carbonate'/exp OR 'lithium chloride'/exp OR 'lithium citrate'/exp OR 'lithium derivative'/exp OR 'lithium fluoride'/exp OR 'lithium gluconate'/exp OR 'lithium hydroxybutyrate'/exp OR 'lithium salt'/exp OR 'lithium sulfate'/exp OR 'neuroleptic agent'/exp OR psychosis/exp/dm_dt OR 'bipolar disorder'/exp/dm_dt OR (lithium* OR carbolit* OR lithane* OR lithonate* OR lithionate* OR antipsychot* OR neuroleptic* OR (major NEXT/1 tranquilizer*) OR aripiprazole* OR bromperidol* OR Chlorpromazine* OR Clozapine* OR Flupenthixol* OR Fluphenazine* OR Haloperidol* OR olanzapine* OR Penfluridol* OR Perazine* OR Perphenazine* OR Pimozide* OR pipamperone* OR Promazine* OR quetiapine* OR Risperidone* OR Sulpiride* OR 'Tiapride Hydrochloride'):ab,ti) AND ('prenatal drug exposure'/exp OR 'prenatal exposure'/exp OR pregnancy/exp OR 'pregnant woman'/exp OR embryotoxicity/exp OR (((prenatal* OR intrauterine* OR maternal OR mother* OR fetal OR foetal OR intra-uterine OR in-utero OR embryo* OR offspring* OR gestation*) NEAR/6 (expos* OR use OR toxic* OR safet* OR medication* OR drug* OR antipsychotic* OR lithium)) OR pregnan* OR embryotoxic*):ab,ti) AND ('child development'/exp OR 'child behavior'/exp OR 'human development'/exp OR behavior/de OR 'animal behavior'/de OR 'adaptive behavior'/exp OR 'adjustment disorder'/exp OR 'adolescent behavior'/exp OR 'coping behavior'/exp OR emotion/exp OR cognition/de OR 'motor activity'/exp OR 'social behavior'/exp OR 'mental development'/exp OR 'behavior disorder'/de OR 'abnormal behavior'/exp OR 'disruptive behavior'/exp OR 'psychomotor disorder'/exp OR 'psychosocial disorder'/exp OR (development* OR neurodevelop* OR behav* OR psychomotor* OR psychosocial* OR emotion* OR fear* OR anxiet* OR well-being):ab,ti) AND (child/exp OR adolescent/exp OR adolescence/exp OR 'child behavior'/de OR pediatrics/exp OR childhood/exp OR 'child development'/de OR 'child growth'/de OR 'child health'/de OR 'child health care'/exp OR 'child care'/exp OR 'childhood disease'/exp OR 'child psychiatry'/de OR 'child psychology'/de OR 'pediatric ward'/de OR 'pediatric hospital'/de OR 'pediatric nursing'/exp OR (adolescen* OR infan* OR baby OR babies OR child* OR kid OR kids OR toddler* OR teen* OR boy* OR girl* OR minors OR underag* OR (under NEXT/1 (age* OR aging)) OR juvenil* OR youth* OR kindergar* OR puber* OR pubescen* OR prepubescen* OR prepubert* OR pediatric* OR paediatric* OR school* OR preschool* OR highschool*):ab,ti)

**MEDLINE (Ovid)**

(lithium/ OR exp "lithium carbonate"/ OR exp "lithium chloride"/ OR exp "lithium Compounds"/ OR exp "Antipsychotic Agents"/ OR exp "Psychotic Disorders"/dt OR "bipolar disorder"/dt OR (lithium* OR carbolit* OR lithane* OR lithonate* OR lithionate* OR antipsychot* OR neuroleptic* OR (major ADJ tranquilizer*) OR aripiprazole* OR bromperidol* OR Chlorpromazine* OR Clozapine* OR Flupenthixol* OR Fluphenazine* OR Haloperidol* OR olanzapine* OR Penfluridol* OR Perazine* OR Perphenazine* OR Pimozide* OR pipamperone* OR Promazine* OR quetiapine* OR Risperidone* OR Sulpiride* OR "Tiapride Hydrochloride").ab,ti.) AND ("Prenatal Exposure Delayed Effects"/ OR exp "Maternal Exposure"/ OR exp pregnancy/ OR "pregnant women"/ OR (((prenatal* OR intrauterine* OR maternal OR mother* OR fetal OR foetal OR intra-uterine OR in-utero OR embryo* OR offspring* OR gestation*) ADJ6 (expos* OR "use" OR toxic* OR safet* OR medication* OR drug* OR antipsychotic* OR lithium)) OR pregnan* OR embryotoxic*).ab,ti.) AND ("Growth and Development"/ OR exp "Human Development"/ OR exp "child behavior"/ OR exp "Child Behavior Disorders"/ OR behavior/ OR "Behavior, Animal"/ OR exp "adaptive behavior"/ OR "adolescent behavior"/ OR "Adaptation, Psychological"/ OR exp emotions/ OR "motor activity"/ OR exp "social behavior"/ OR exp "Social Behavior Disorders"/ OR exp "Neurobehavioral Manifestations"/ OR "Mental Disorders Diagnosed in Childhood"/ OR exp "psychomotor disorders"/ OR "Adjustment Disorders"/ OR (development* OR neurodevelop* OR behav* OR psychomotor* OR psychosocial* OR emotion* OR fear* OR anxiet* OR well-being).ab,ti.) AND (exp child/ OR exp infant/ OR adolescent/ OR exp pediatrics/ OR exp Child Health Services/ OR Hospitals, Pediatric/ OR (adolescen* OR infan* OR baby OR babies OR child* OR kid OR kids OR toddler* OR teen* OR boy* OR girl* OR minors OR underag* OR (under ADJ (age* OR aging)) OR juvenil* OR youth* OR kindergar* OR puber* OR pubescen* OR prepubescen* OR prepubert* OR pediatric* OR paediatric* OR school* OR preschool* OR highschool*).ab,ti.)

**PsycINFO (Ovid)**

(lithium/ OR exp "lithium carbonate"/ OR exp "Neuroleptic Drugs"/ OR exp "Psychosis"/dt OR "bipolar disorder"/dt OR (lithium* OR carbolit* OR lithane* OR lithonate* OR lithionate* OR antipsychot* OR neuroleptic* OR (major ADJ tranquilizer*) OR aripiprazole* OR bromperidol* OR Chlorpromazine* OR Clozapine* OR Flupenthixol* OR Fluphenazine* OR Haloperidol* OR olanzapine* OR Penfluridol* OR Perazine* OR Perphenazine* OR Pimozide* OR pipamperone* OR Promazine* OR quetiapine* OR Risperidone* OR Sulpiride* OR "Tiapride Hydrochloride").ab,ti.) AND ("Prenatal Exposure"/ OR exp exp pregnancy/ OR (((prenatal* OR intrauterine* OR maternal OR mother* OR fetal OR foetal OR intra-uterine OR in-utero OR embryo* OR offspring* OR gestation*) ADJ6 (expos* OR "use" OR toxic* OR safet* OR medication* OR drug* OR antipsychotic* OR lithium)) OR pregnan* OR embryotoxic*).ab,ti.) AND ("Development"/ OR "Animal Development"/ OR exp "Delayed Development"/ OR exp "Human Development"/ OR exp "Psychological Development"/ OR exp "Behavior Problems"/ OR behavior/ OR "Adaptive Behavior"/ OR "Attachment Behavior"/ OR "Classroom Behavior"/ OR "Coping Behavior"/ OR exp "Exploratory Behavior"/ OR exp "Social Behavior"/ OR exp emotions/ OR exp "Motor Performance"/ OR "Behavior Disorders"/ OR exp "Psychomotor Development"/ OR "Adjustment Disorders"/ OR (development* OR neurodevelop* OR behav* OR psychomotor* OR psychosocial* OR emotion* OR fear* OR anxiet* OR well-being).ab,ti.) AND (140.ag. OR 160.ag. OR 180.ag. OR 200.ag. OR exp pediatrics/ OR exp Hospitals, Pediatric/ OR (adolescen* OR infan* OR baby OR babies OR child* OR kid OR kids OR toddler* OR teen* OR boy* OR girl* OR minors OR underag* OR (under ADJ (age* OR aging)) OR juvenil* OR youth* OR kindergar* OR puber* OR pubescen* OR prepubescen* OR prepubert* OR pediatric* OR paediatric* OR school* OR preschool* OR highschool*).ab,ti.)

**Cochrane**

((lithium* OR carbolit* OR lithane* OR lithonate* OR lithionate* OR antipsychot* OR neuroleptic* OR (major NEXT/1 tranquilizer*) OR aripiprazole* OR bromperidol* OR Chlorpromazine* OR Clozapine* OR Flupenthixol* OR Fluphenazine* OR Haloperidol* OR olanzapine* OR Penfluridol* OR Perazine* OR Perphenazine* OR Pimozide* OR pipamperone* OR Promazine* OR quetiapine* OR Risperidone* OR Sulpiride* OR 'Tiapride Hydrochloride'):ab,ti) AND ((((prenatal* OR intrauterine* OR maternal OR mother* OR fetal OR foetal OR intra-uterine OR in-utero OR embryo* OR offspring* OR gestation*) NEAR/6 (expos* OR use OR toxic* OR safet* OR medication* OR drug* OR antipsychotic* OR lithium)) OR pregnan* OR embryotoxic*):ab,ti) AND ((development* OR neurodevelop* OR behav* OR psychomotor* OR psychosocial* OR emotion* OR fear* OR anxiet* OR well-being):ab,ti) AND ((adolescen* OR infan* OR baby OR babies OR child* OR kid OR kids OR toddler* OR teen* OR boy* OR girl* OR minors OR underag* OR (under NEXT/1 (age* OR aging)) OR juvenil* OR youth* OR kindergar* OR puber* OR pubescen* OR prepubescen* OR prepubert* OR pediatric* OR paediatric* OR school* OR preschool* OR highschool*):ab,ti)

**Web of Science**

TS=(((lithium* OR carbolit* OR lithane* OR lithonate* OR lithionate* OR antipsychot* OR neuroleptic* OR (major NEAR/1 tranquilizer*) OR aripiprazole* OR bromperidol* OR Chlorpromazine* OR Clozapine* OR Flupenthixol* OR Fluphenazine* OR Haloperidol* OR olanzapine* OR Penfluridol* OR Perazine* OR Perphenazine* OR Pimozide* OR pipamperone* OR Promazine* OR quetiapine* OR Risperidone* OR Sulpiride* OR "Tiapride Hydrochloride")) AND ((((prenatal* OR intrauterine* OR maternal OR mother* OR fetal OR foetal OR intra-uterine OR in-utero OR embryo* OR offspring* OR gestation*) NEAR/6 (expos* OR use OR toxic* OR safet* OR medication* OR drug* OR antipsychotic* OR lithium)) OR pregnan* OR embryotoxic*)) AND ((development* OR neurodevelop* OR behav* OR psychomotor* OR psychosocial* OR emotion* OR fear* OR anxiet* OR well-being)) AND ((adolescen* OR infan* OR baby OR babies OR child* OR kid OR kids OR toddler* OR teen* OR boy* OR girl* OR minors OR underag* OR (under NEAR/1 (age* OR aging)) OR juvenil* OR youth* OR kindergar* OR puber* OR pubescen* OR prepubescen* OR prepubert* OR pediatric* OR paediatric* OR school* OR preschool* OR highschool*)))

**Google Scholar**

lithium|antipsychotic|antipsychotics|neuroleptic|neuroleptics "prenatal|intrauterine|fetal|foetal|uterine|utero exposure" development|neurodevelopment|behavior|psychomotor|psychosocial|emotions|fear|anxiety adolescent|infant|infants|child|children
