# Supplementary material for: Long-term neurodevelopmental consequences of intrauterine exposure to lithium and antipsychotics: a systematic review and meta-analysis
Source: Eur Child Adolesc Psychiatry. 2018 Jun 11;27(9):1209–30. doi: 10.1007/s00787-018-1177-1 (PMC6133089; doi:10.1007/s00787-018-1177-1)
Supplement: Supplementary file 2 — Supplementary material 2 (DOCX 23 kb) [file 787_2018_1177_MOESM2_ESM.docx]

**Long-term neurodevelopmental consequences of intrauterine exposure to lithium and antipsychotics: a systematic review and meta-analysis**

Eline M. P. Poels^1^, Lisanne Schrijver^1^, Astrid M. Kamperman^1^, Manon H.J. Hillegers^2^, Witte J. G. Hoogendijk^1^, Steven A. Kushner^1^, Sabine J. Roza^1^

^1^ Department of Psychiatry, Erasmus University Medical Center, ’s-Gravendijkwal 230, 3015 CE, Rotterdam, The Netherlands

^2^ Department of Child and Adolescent Psychiatry, Erasmus University Medical Center, Rotterdam, The Netherlands

Supplementary material 2

Table 1: Characteristics of case studies on the neurodevelopmental outcome after intrauterine exposure to lithium

| **Author (year)** | **Sample size** | **Medication (daily dosage)** | **Treatment indication** | **Follow-up time** | **Results** |
| --- | --- | --- | --- | --- | --- |
| Morrel (1983) | 1 | Lithium (1200mg) + chlorpromazine (50mg) | Bipolar disorder | 1 year | Delayed motor development and a concomitant squint. |
| Frassetto (2002) | 1 | Lithium, Cyamemazine, Olanzapine, Venlafaxine (n.r.) | Bipolar disorder | 3.5 months | Normal psychomotor development. |
| Kozma (2005) | 1 | Lithium (900-1350mg) | Bipolar disorder | 13 months | At 3 weeks: hypotonic, decreased neonatal reflexes, decreased social interaction.  At 13 months: normal scores on the BSID, slightly decreased muscle tone. |
| Burt (2010) | 1 | Lithium (1125-1350mg),  Olanzapine (10-20mg),  Fluoxetine (40-60mg) | Bipolar disorder | 29 months | Delay of gross motor development up to the age of 29 months. |
| Bogen (2012) | 4 | Lithium (varied) | Bipolar disorder | 4 months – 1 year | One child with gross and fine motor delay in the first year. |

*n.r.* not reported, *BSID* Bayley Scale of Infant Development

Table 2: Characteristics of case studies on the neurodevelopmental outcome after intrauterine exposure to antipsychotics

| **Author (year)** | **Sample size** | **Medication (daily dosage)** | **Treatment indication** | **Follow-up time** | **Results** |
| --- | --- | --- | --- | --- | --- |
| Hammond (1970) | 1 | Chlorpromazine (1800 mg) | Schizoaffective disorder | 6 months | Normal development. |
| O’Connor (1981) | 1 | Chlorpromazine (1200mg),  Fluphenazine (100mg) | Schizophrenia | 15 months | First months: high irritability and motor deficits, 15 months: normal development. |
| Tanaka (1990) | 2 | Haloperidol (1.5-2.5mg) | Psychotic disorders | 2 – 5 months | Normal physical and mental development. |
| Barnas (1994) | 1 | Clozapine (50-100mg) | Schizophrenia | 6 months | Normal psychomotor development. |
| Stingl (2000) | 1 | Olanzapine (10mg) | Schizophrenia | 11 months | Impaired motor development at 7 months, normal development at 11 months. |
| Ratnayake (2002) | 2 | Risperidone (2-6 mg) | Schizophrenia | 9 – 12 months | No developmental abnormalities. |
| Tenyi (2002) | 1 | Quetiapine (150-300mg) | Schizophrenia | 6 months | Normal development. |
| Karakula (2004) | 1 | Clozapine (200mg) | Schizophrenia | 7 months | Encephalopathy after birth with convulsions and floppy infant syndrome, at 7 months delayed development. |
| Dabbert (2006) | 1 | LAI risperidone (25mg/2weeks) | Schizophrenia | 2,5 years | Minor retardation in motor development at 2 years but within normal range. |
| Mendhekar (2006) | 1 | Aripiprazole (10mg) | Schizoaffective disorder | 6 months | Normal achievement of milestones. |
| Cabuk (2007) | 1 | Quetiapine (1200mg), Haloperidol (15mg) | Bipolar disorder | 80 days | Normal development. |
| Kim (2007) | 1 | Risperidone (25mg/2weeks) | Schizophrenia | 8 months | Normal development reported. |
| Klier (2007) | 1 | Quetiapine (300 mg), Venlafaxine (75 mg),  Trazodone (150 mg) | Bipolar disorder | 1 year | Normal achievement of milestones. |
| Mendhekar (2007) | 1 | Clozapine (100mg) | Schizophrenia | 5 years | Delayed speech up until 5 years of age. |
| Aichhorn (2008) | 1 | Olanzapine (15 mg) | Schizophrenia | 6 months | Normal development. |
| Mendhekar (2008) | 2 (1 mother) | Risperidone (2-3 mg) | Schizophrenia | 18 – 36 months | No evidence of neurodevelopmental delay. |
| Mendhekar (2011) | 3 (1 mother) | 1^st^+2^nd^ pregnancy: haloperidol (7,5-10 mg), 3^rd^ pregnancy: haloperidol (15 mg) + risperidone (2mg) | Schizophrenia | 16 months – 8 years | Normal developmental milestones and educational attainments. |
| Werremeyer (2009) | 1 | Ziprasidone (40mg) Citalopram (60mg) | Psychotic depression | 6 months | Normal development. |
| Wichman (2009) | 16 | Atypical antipsychotics (varied) | Schizophrenia, schizoaffective disorder, MDD, bipolar disorder | 3 years | Two infants with behavioral concerns. One infant with speech delay. |
| Rowe (2012) | 1 | Olanzapine (15-20mg), Promethazine (100mg), Lithium (1600mg),  Diazepam (5mg) | Bipolar disorder | 5 months | Normal development. |
| Janjic (2013) | 2 (1 mother) | LAI zuclopentixol (1^st^ pregnancy: 400mg/2weeks,  2^nd^ pregnancy: 200mg/month) | Schizophrenia | 6months - 3years | Normal development. |
| Stiegler (2014) | 1 | Olanzapine (15mg) | Schizophrenia | 20 months | Normal development. |
| Gentile (2015) | 1 | Quetiapine (400 mg), Trazodone (200 mg)  Lorazepam (7.5 mg)  Mirtazapine(30 mg)  Flurazepam (30 mg) | Borderline personality disorder | 4 months | No developmental delay. |
| Kenar (2015) | 1 | Olanzapine (10 mg) Sertraline (100 mg) Quetiapine (400 mg) Chlorpromazine (100mg) | Generalized anxiety disorder | 2 years | Normal motor and mental development. |
| Rodriguez (2017) | 1 | LAI paliperidone (100mg/4weeks) | Schizoaffective disorder | 1 year | Normal psychomotor development. |
| McCauley (2014) | 5 | Olanzapine, Risperidone, Quetiapine, Haloperidol (n.r.) | Serious mental illness | 1 year | 4 children with normal development. One child had delayed motor development. |

*MDD* major depressive disorder, *n.r.* not reported, *LAI* long acting injectable
