# Supplementary figures and images for: Long-term neurodevelopmental consequences of intrauterine exposure to lithium and antipsychotics: a systematic review and meta-analysis
Source: Eur Child Adolesc Psychiatry. 2018 Jun 11;27(9):1209–30. doi: 10.1007/s00787-018-1177-1 (PMC6133089; doi:10.1007/s00787-018-1177-1)

a.

### Risk of bias: Lithium

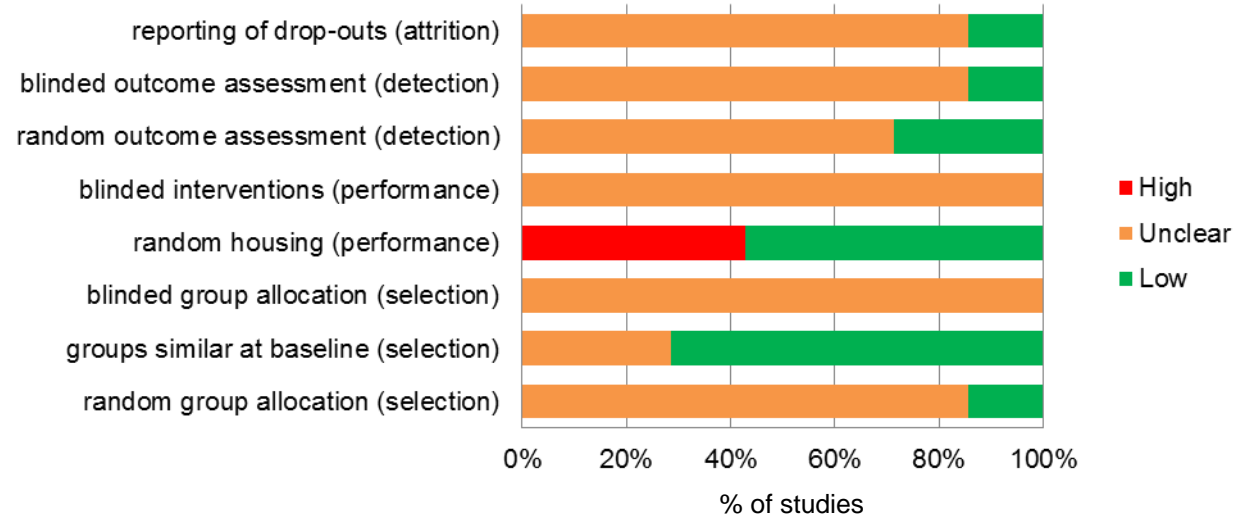

b.

### Risk of bias: Antipsychotic

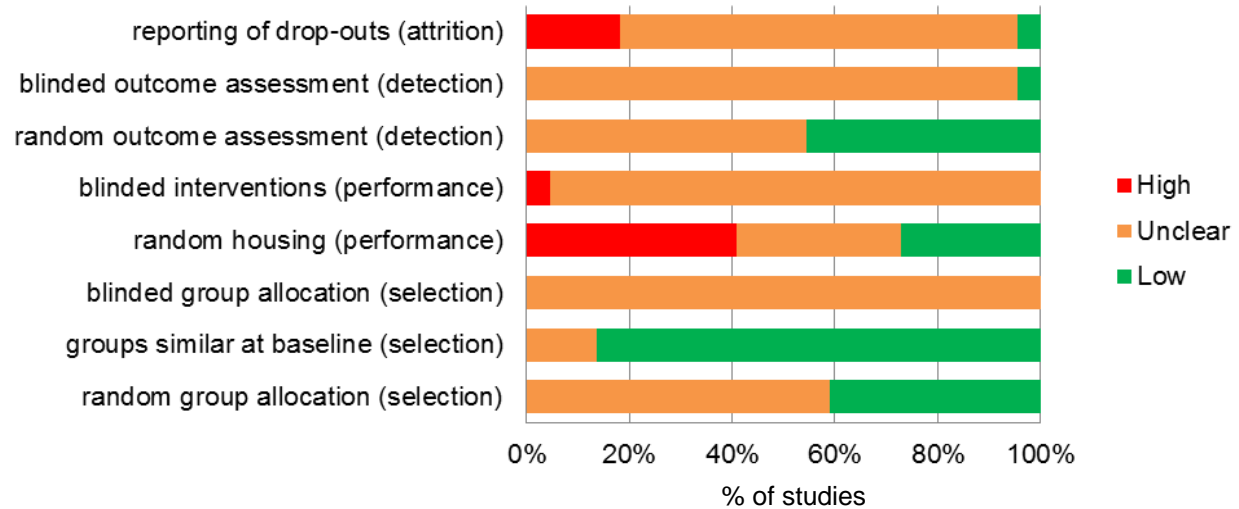

Supplement: Supplementary file 3 — Supplementary material 3 (PDF 41 kb) [file 787_2018_1177_MOESM3_ESM.pdf]
